# Mental Health Promotion Through Nursing Interventions Focused on Anxiety, Sleep and Well‐Being: A Scoping Review

**DOI:** 10.1111/inm.70254

**Published:** 2026-04-08

**Authors:** Louise Constancia de Melo Alves Silva, Samia Valeria Ozorio Dutra, Barbara Fernandes Ferreira, Yenifer Lizeth Gañan Rojas, Kauanny Vitoria Gurgel dos Santos, Carlos Jordão Assis de Silva, Hylarina Maria Montenegro Diniz Silva, Kátia Regina Barros Ribeiro, Daniele Vieira Dantas, Rodrigo Assis Neves Dantas

**Affiliations:** ^1^ Postgraduate Program in Nursing Federal University of Rio Grande do Norte Natal RN Brazil; ^2^ School of Nursing and Dental Hygiene, University of Hawaii Manoa Hawaii USA; ^3^ Department of Nursing Federal University of Rio Grande Do Norte Natal RN Brazil

**Keywords:** adult, aged, anxiety, health services, nursing care, sleep, well‐being

## Abstract

Anxiety is more prevalent than other mental disorders and maintains a bidirectional relationship with sleep disturbances, negatively affecting well‐being and overall mental health. This scenario underscores the need to strengthen and qualify nursing practice in addressing these outcomes. This study aimed to map how nursing has developed care for adults and older people through interventions related to anxiety, sleep quality and well‐being. A scoping review was conducted in accordance with the PRISMA‐ScR checklist. Searches were performed between November and December 2025 across 11 data sources: CINAHL, Cochrane Library, LILACS, MEDLINE/PubMed, SciELO, Scopus, EBSCO Open Dissertations, Web of Science, CAPES Catalogue of Theses and Dissertations, ScienceDirect and Google Scholar, in addition to reference list screening. Sixty‐four studies were included. Although the search covered the period from 2010 to 2025, eligible publications were identified between 2011 and 2025, predominantly clinical trials conducted in Iran and Turkey. Thirty‐one occurrences of interventions were identified, representing different modes of application of a smaller set of nursing‐implementable strategies targeting anxiety, sleep and well‐being. Integrative and complementary therapies predominated, including aromatherapy, music therapy, reflexology, acupressure, therapeutic touch, massage, relaxation techniques, Reiki and yoga. Other interventions comprised spiritual support, environmental adjustments (lighting, noise, temperature control), guided imagery, group counselling and self‐care programmes, reinforcing nursing's holistic approach. Sleep quality was the most investigated outcome, mainly through environmental control during hospitalisation. However, considering sleep, well‐being and anxiety, multicentre studies, further investigation into spirituality and the nursing process and research prioritising well‐being and anxiety disorders as primary outcomes are still needed.

## 1 Introduction

Mental disorders, including anxiety disorders, are considered among the greatest current global challenges, associated with high morbidity, disability and premature mortality. According to the World Health Organization (WHO), approximately 4.4% of the world's population currently suffers from some type of anxiety disorder, and approximately 359 million people were living with anxiety disorders in 2021, making them more common than any other type of mental disorder (World Health Organization 2025a).

According to NANDA International (2021–2023), the nursing diagnosis ‘Anxiety’ is defined as ‘A vague, uneasy feeling of discomfort or dread accompanied by an autonomic response; the source is often non‐specific or unknown to the individual; a feeling of apprehension caused by anticipation of danger’ (Herdman, Kamitsuru and Lopes Herdman et al. 2021). Most of the time, anxiety is understood as a normal manifestation of an affective state, which allows the individual to remain alert to dangers and survive threats or even adapt to the unknown (World Health Organization [WHO] 2025b).

Anxiety disorders are characterised by intense and persistent symptoms that exceed everyday anxiety, generating suffering and life impairments, as many individuals cease to perform their routine activities due to the fear of panic attacks or associated symptoms (Galarça et al. 2025). Meanwhile, well‐being is defined as a subjective and multidimensional state that involves a positive perception of one's own life, including physical, emotional, psychological and social aspects (Yong et al. 2025).

High levels of anxiety are associated with lower satisfaction and emotional stability, acting as a predictor of reduced well‐being and reinforcing the importance of therapeutic approaches that strengthen this aspect in the care of individuals with anxiety symptoms (Yong et al. 2025).

Anxiety is also directly linked to sleep disturbances in a bidirectional relationship, in which anxiety worsens sleep and poor‐quality sleep intensifies anxious symptoms, forming a feedback loop. In this context, studies suggest that this relationship may be mediated by the dysregulation of the hypothalamic–pituitary–adrenal (HPA) axis, as insufficient sleep increases cortisol production and compromises the adaptive response to stress. Furthermore, the loss of a single night's sleep increases amygdala reactivity and reduces connectivity with the medial prefrontal cortex, similar to findings in anxiety disorders (Cox and Olatunji 2020).

Regarding the actions of health professionals facing individuals with anxious symptoms, the central role of nursing stands out, as it organises and executes care holistically and continuously 24 h a day. In this context, recognising specific interventions within the nurse's competence contributes to strengthening their role in anxiety care across different healthcare settings (Pereira et al. 2023).

Furthermore, within the nursing process, it is the nurse's responsibility to approach the individual holistically and use strategies that minimise anxiety related to both the disease and the involved treatments. To this end, studies also indicate the need to expand and qualify this practice through new forms of care that ensure patient integrity and address dimensions that are often overlooked by other health professionals (Jesus et al. 2025).

Given the above, considering the relevance of care for the anxious patient and its repercussions on sleep and well‐being, a study that identifies research involving nursing interventions targeted at these three outcomes is justified. A previous search of the literature revealed no scoping review to date that associates nursing care directed at anxiety with explicit outcomes of sleep quality and well‐being in the adult and elderly population, which reinforces the originality and relevance of the present proposal.

## 2 Objective

The objective of this study is to map how nursing has developed care for adults and older people through interventions related to anxiety, sleep quality and well‐being.

## 3 Methods

To address the objective of this study, a scoping review was chosen, as it is a methodology capable of broadly mapping the existing literature on nursing interventions related to anxiety, sleep and well‐being. A scoping review provides an overview of available knowledge, identifies gaps, clarifies concepts and helps understand the variety of approaches used, resulting in a comprehensive and organised synthesis of existing evidence (Arksey and O'Malley 2005).

This review was conducted in accordance with the recommendations established by the Preferred Reporting Items for Systematic Reviews and Meta‐analysis Protocols‐extension for Scoping Reviews (PRISMA‐ScR) (Tricco et al. 2018) and followed the methodological guidelines proposed by the Joanna Briggs Institute (JBI), based on the work of Arksey and O'Malley (2005) and expanded by Peters et al. (2020).

The scoping review protocol was registered on the Open Science Framework on 8 November 2025 and is available for consultation at: https://osf.io/jy5sg/overview?view_only=ef2cfdea05a640bc834f001532d8a062.

### 3.1 Research Question

To formulate the research question for this scoping review, the ‘Population, Concept, and Context’ (PCC) mnemonic recommended by the JBI was utilised. Consequently, the PCC for this review was defined as follows: (P) Population: adults receiving nursing care for anxiety, sleep and/or well‐being; (C) Concept: assessment strategies, diagnoses, interventions (including non‐pharmacological ones) and nursing outcomes focused on anxiety and/or sleep quality and well‐being; and (C) Context: any nursing practice setting.

Thus, the following research question was proposed: ‘How has nursing provided care related to anxiety, sleep quality and well‐being in adults and the elderly?’

### 3.2 Eligibility Criteria

Based on the adopted PCC strategy, inclusion criteria were defined as: adults and/or older people in various nursing practice settings, where studies addressed nursing care or interventions related to anxiety and/or included interventions focused on sleep quality and well‐being. Studies that presented other associated outcomes, such as depression or pain, in addition to these primary outcomes, were also included.

Furthermore, the review considered primary quantitative, qualitative or mixed‐methods studies; research protocols; methodological studies; care guidelines; scoping and systematic reviews relevant to the mapping; case studies; theses; and dissertations (to mitigate publication bias and capture non‐indexed evidence). The search included publications between 2010 and 2025 to encompass contemporary evidence of integrated nursing practices and included studies published in Portuguese, English and Spanish.

Exclusion criteria included: studies where the nursing role was not clearly identified; those focused on the care of other professionals or exclusively medical/pharmacological care; editorials; and research involving paediatric, obstetric and/or maternal‐child populations.

### 3.3 Search Strategy

Eleven data sources were used for this review: CINAHL, Cochrane Library, Latin American and Caribbean Health Sciences Literature (LILACS), Medical Literature Analysis and Retrieval System Online (MEDLINE)/PubMed, Scientific Electronic Library Online (SciELO), Scopus, EBSCO Open Dissertations, Web of Science, CAPES Catalogue of Theses and Dissertations, ScienceDirect and Google Scholar.

Indexed descriptors from Medical Subject Headings (MeSH) were used, namely: ‘Adult’; ‘Aged’; ‘Patients’; ‘Anxiety’; ‘anxiety disorders’; ‘Nursing Care’; ‘Nursing Process’; ‘Patient Care Planning’; ‘Sleep’; ‘Sleep Quality’; ‘Well‐Being’; and ‘Health Services’. For Portuguese‐language data sources, the corresponding indexed descriptors from Health Sciences Descriptors (DeCS) were employed. Terms were combined using the Boolean operators ‘AND’ and ‘OR’. The search strategy was adjusted according to the specificities of each database and is presented in Table 1.

**TABLE 1:** Search strategies used in the data sources.

Sources	Syntax	Filters
CINAHL	adults OR aged AND patients AND anxiety OR anxiety disorders AND nursing care OR nursing process OR patient care planning AND sleep OR sleep quality AND well‐being AND health services	Full Text; English, Portuguese and Spanish; Aged; Middle aged; Adult: 19–44 years; 2010–2025
Cochrane Library	“adults”, “aged” in Title Abstract Keyword AND “anxiety”, “anxiety disorders”, “patients” in Title Abstract Keyword AND “nursing care”, “nursing”, “patient care planning” in Title Abstract Keyword AND “sleep”, “sleep quality” in Title Abstract Keyword AND “well‐being”, “health services” in Title Abstract Keyword ‐ (Word variations have been searched)	2010–2025
LILACS	(Adult) OR (Aged) AND (Patients) AND (Anxiety) AND (Nursing Care) AND (Sleep) AND (Well‐Being) AND (Health Services)	None
MEDLINE/PubMed	(((((((((((adults) OR (aged)) AND (patients)) AND (anxiety)) OR (anxiety disorders)) AND (nursing care)) OR (nursing process)) OR (patient care planning)) AND (sleep)) OR (sleep quality)) AND (well‐being)) AND (health services)	Full text; 2010–2025
SciELO	adults OR aged AND patients AND anxiety OR anxiety disorders AND nursing care OR nursing process OR patient care planning AND sleep OR sleep quality AND well‐being AND health services	2010–2025
Scopus	ALL(“Adult” OR “Aged”) AND (“Anxiety” OR “Anxiety Disorders”) AND (“Nursing Care” OR “Nursing Process” OR “Patient Care Planning”) AND (“Sleep” OR “Sleep Quality”) AND (“Well‐Being”) AND (“Health Services”)	2010–2025; English and Spanish; Nursing
EBSCO Open Dissertations	adults OR aged AND patients AND anxiety OR anxiety disorders AND nursing care OR nursing process OR patient care planning AND sleep OR sleep quality AND well‐being AND health services	2010–2025
Web of Science	adults (Topic) or aged (All Fields) and patients (All Fields) and anxiety (All Fields) or anxiety disorders (All Fields) and Nursing Care (All Fields) or Nursing Process (All Fields) or Patient Care Planning (All Fields) and Sleep (All Fields) or Sleep Quality (All Fields) and Well‐Being (All Fields) and Health Services (All Fields)	2010–2025; Categories: Nursing; English, Portuguese and Spanish; All open access, Area: Nursing
Thesis and Dissertation Catalogue (CAPES)	Anxiety, Nursing care, Sleep, Well‐being	None
ScienceDirect	adults OR aged AND patients AND anxiety AND nursing care OR nursing process AND sleep AND well‐being AND health services	2010–2025; Review articles; Research articles; Area: Nursing and Health Professions; English and Spanish; Open access and Open archive
Google Scholar	anxiety disorders AND nursing care OR nursing process OR patient care planning AND sleep OR sleep quality AND well‐being AND health services	2010–2025

### 3.4 Data Collection

Screening of titles and abstracts across the data sources was conducted on 10 and 11 November 2025, according to the defined descriptors and operators. All results were transferred to the Rayyan software, where two reviewers independently analysed titles and abstracts between 15 November and 2 December 2025, selecting eligible studies for full‐text reading.

Following the initial selection, a reverse search was performed by examining the reference lists of the studies included in the initial screening to identify additional relevant publications, following the same methodological rigour as the previous search and selection.

### 3.5 Data Extraction and Analysis

Data extraction was carried out through a full reading of the selected articles, and all information was systematised into a database created in Microsoft Excel, available in Data S1 section for full consultation.

The database included bibliographic variables such as authors, year of publication, country or region of the study, language and study type. Descriptive variables regarding the characteristics of the study population were also recorded, considering the participants' profile and the healthcare context, as well as information relating to the nursing role, including training, function and duties performed, where described.

Instruments used to assess anxiety, sleep quality and well‐being were identified where present. Additionally, any described nursing diagnoses were extracted, as well as the interventions performed, which included both standardised actions within the nursing process and non‐pharmacological interventions, such as integrative and complementary practices.

Interventions described in the articles were extracted according to the structural elements of the Template for Intervention Description and Replication (TIDieR), considering who performed it, what was done, how, where, when, duration, adaptations and fidelity (Hoffmann et al. 2014). Outcomes related to anxiety relief, sleep quality and well‐being were also recorded, with a description of the results obtained and the existence of monitoring or follow‐up after the interventions.

Where available, barriers and facilitators related to the implementation of the interventions described in the studies were mapped. Finally, knowledge gaps were identified and subsequently organised according to the PAGER model (Bradbury‐Jones et al. 2022), which will be presented in the results, allowing for a deeper understanding of the theme and guiding future recommendations.

## 4 Results

**FIGURE 1:**
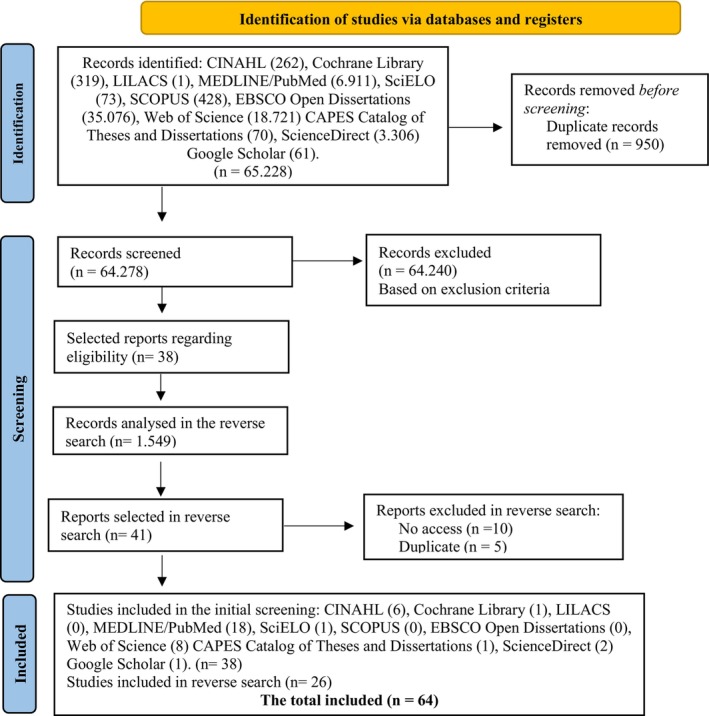
Search flowchart adapted from the Preferred Reporting Items for Systematic Reviews and Meta‐Analyses extension for Scoping Reviews (PRISMA‐ScR).

Figure 1 presents the PRISMA flow diagram (Page et al. 2021) of the study selection process, tracing the journey from the identification of records in the selected databases to the final sample of 64 articles, following the application of eligibility criteria and the removal of duplicates.

Given the large number of included studies, the authors have provided a database with the complete extraction of the selected articles as supporting information. Consequently, this section presents a general overview of the results and the main findings of the review without individually citing all 64 studies, the details of which can be consulted in the supporting information (see Data S1: Database).

The search covered the period from 2010 to 2025; however, no eligible studies published in 2010 were identified. Therefore, the included studies cover the period from 2011 to 2025. Notable peaks occurred in 2017 and 2021, with six (9.37%) articles each and 2022, with seven (10.93%) articles; other years showed a balanced distribution. In terms of geographical distribution, a higher concentration of research was observed in Iran, with 17 (26.56%) articles, and Turkey, with 11 (17.18%) articles, covering the various interventions presented in this study. Other identified studies originated from China, Taiwan, the United States, Spain, India, South Korea, Egypt, Indonesia, Brazil, Jordan, Canada, the Czech Republic, Chile, Italy, Colombia and Denmark (see Data S1: Database).

Regarding the study designs selected, the majority were clinical trials (*n* = 38; 59.37%), followed by quasi‐experimental studies (*n* = 14; 21.87%), systematic reviews (*n* = 5; 7.81%) and umbrella reviews (*n* = 2; 3.12%). The remaining designs each accounted for a single study (1.56%) and included a case report, a methodological study, a descriptive‐analytical survey, a dissertation and a cohort study (see S1. Database).

In general, there was a predominance of improvement in parameters related to sleep and anxiety among the included studies (see Data S1: Database). Sleep improvement was reported in 32 studies (50%), while anxiety reduction was described in 25 studies (39%), often accompanied by mention of statistical significance. Among the interventions identified, aromatherapy stood out for its recurrence in the set of studies analysed, as did acupressure and reflexology. Although the designs and contexts vary, the findings show a consistent trend of positive effects, especially on sleep, indicating convergence of results regarding the improvement of these mental health parameters.

Of the 52 clinical trials and quasi‐experimental studies, 15 different types of healthcare services were identified. Interventions were most frequently conducted in Intensive Care Units (ICU), with 18 (28.12%) articles, followed by cardiac care units, with six (9.37%) studies (see Data S1: Database). The remaining work was distributed across sectors such as haemodialysis, burn units, home care, general wards, palliative care, operating theatres and other specific contexts.

**FIGURE 2:**
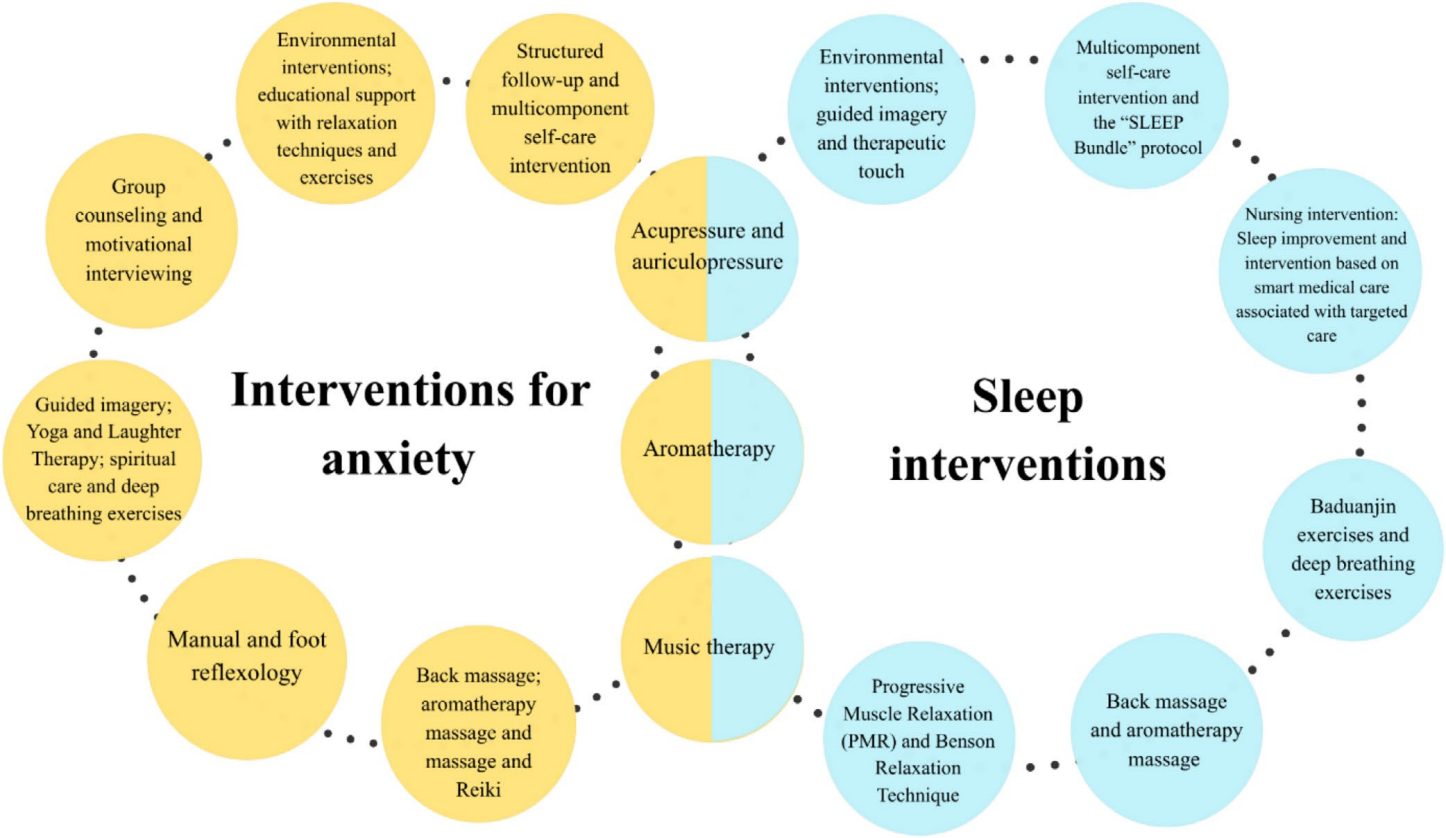
Summary of nursing interventions evaluated in 52 studies, including clinical and quasi‐experimental trials, with an emphasis on anxiety and sleep.

By analysing the included clinical and quasi‐experimental trials, this review synthesised nursing interventions related to anxiety and the quality and promotion of sleep (Figure 2). However, as few studies evaluated well‐being as an outcome, this result was not included in the figure.

The studies testing interventions for sleep, anxiety and well‐being utilised various scales, ranging from validated and adapted tools to those developed by the authors themselves. Table 2 collates the most frequently used scales in the intervention research for the outcomes analysed.

**TABLE 2:** Main scales used in studies for anxiety, sleep and well‐being.

Outcomes	Main scales	Number (%)
Anxiety	State–Trait Anxiety Inventory (STAI–I e STAI–II)	20 (37.73)
	Hospital Anxiety and Depression Scale (HADS)	4 (7.54)
	Visual Analog Scale (VAS‐A)	4 (7.54)
Sleep	Pittsburgh Sleep Quality Index (PSQI)	13 (24.52)
	Richards‐Campbell Sleep Questionnaire (RCSQ)	10 (18.86)
	Verran Snyder‐Halpern Sleep Scale (VSH)	3 (5.66)
	St. Mary's Hospital Sleep Questionnaire (SMHSQ)	3 (5.66)
Well‐being	HERO Wellness Scale	1 (1.88)
	Medical Outcomes Study 36‐Item Short‐Form Health Survey (SF‐36)	
	Arabic Scale of Happiness (ASH)	

*Source:* S1. Database.

Beyond the data, the primary highlight of this review is found in Table 3, which brings together all interventions, their modes of application, target populations and outcomes, systematised according to the TIDieR items used during data extraction. The review identified 31 occurrences of interventions, representing different applications of a smaller set of interventions, implementable by nursing, focused on anxiety, sleep and/or well‐being. Sleep was one of the most explored outcomes; as evidenced in the table, interventions varied between integrative and complementary therapies, environmental interventions and specific protocols. Notably, some studies also evaluated other outcomes (pain, depression, spirituality, vital signs, etc.), though data extraction was restricted to the primary outcomes of interest.

**TABLE 3:** Nursing interventions focused on anxiety, sleep and well‐being, identified in the 52 studies, including clinical and quasi‐experimental trials.

Components of the intervention	Implementation of the intervention	Patient background	Main outcomes
Aromatherapy—3 articles (5.76%)			
Lavender oil on cotton wool/gauze/wet wipe	Apply 2 to 7 drops of oil to the material of choice, 10–30 cm away from the patient, with an inhalation time of 20 min	Patients in: Coronary care unit; burn treatment centre and candidates for cardiac and abdominal surgery	Two of the three studies showed improvement in anxiety. All studies improved sleep
Aromatherapy with a combination of two or more oils—2 articles (3.84%)			
Lavender + bergamot oil; lavender + lemon on pillows	Apply 3 drops of 100% pure oil to the chosen material, 30 cm below the patient's nose and inhale for 20 min	Patients in coronary care units and hospitalized in surgical intensive care units	One of the two studies reduced anxiety and both improved sleep
Aromatherapy with Rosa damascena oil—1 article (1.92%)			
Damask rose oil on absorbent napkin	Application of 5 drops of 40% oil to the material of choice, attached to the collar of the patient's clothing, 20 cm from the nose. The napkin remained in place for 8 h	Adults admitted to burn units	Significant reduction in anxiety levels and improved sleep quality
Aromatherapy using external patches (stickers or portable sachets)—1 articles (1.92%)			
Essential oils in two versions: lavender‐sandalwood and orange‐peppermint on external tabs	External aromatherapy tabs containing 0.2 mL of essential oils were attached to the patient's gown for 64 min	Women undergoing breast biopsies	Significant reduction in state anxiety
Music therapy—6 articles (11.53%)			
Songs played on MP3; speaker; CD player; live music; group singing; ensemble instruments and headphones associated with sleep eye mask	Sounds of nature; delta wave music; excerpts from the Goldberg Variations; pop, jazz, classical or rock music; instrumental music with a gentle rhythm; and popular Chinese and Taiwanese songs. The interventions lasted between 20 and 52 min	Adults undergoing outpatient surgery; Intensive Care Unit (ICU); psychiatric ward; women referred for outpatient hysteroscopy and patients undergoing coronary angioplasty	Significant improvement in sleep quality and reduction in anxiety levels
Manual reflexology—2 articles (3.84%)			
Use warm water/sesame oil	In one of the articles, general hand massage was performed eight times, followed by stimulation of the six reflexology points, repeated 14 times. In the second, patients also received general hand massage, followed by 2 min of stimulation at the three reflexology points (pituitary gland, heart and solar plexus) for 20 min	Patients undergoing coronary angiography for the first time	Significant decrease in anxiety levels in patients in both studies
Foot reflexology—2 articles (3.84%)			
Lubricating cream in one article and warm water with sunflower oil in another article	In one of the articles, patients received a general foot massage, followed by stimulation of the three reflexology areas (solar plexus, pituitary gland and heart) for 30 min. In the second article, lubricating cream was applied to the left foot for 1 min, followed by reflexology massage on the sole of the foot for 20 min	Patients who have undergone cardiac surgery and coronary angiography	Significant decrease in anxiety levels
Acupressure—2 articles (3.84%)			
In one of the articles, the application was made at the following points: Heart 7 (H7), Pericardium 6 (MC6 5 PC6), Gallbladder 20 (GB20) and Stomach 6 (ST6). In the other, acupressure was applied at five different solar points: forehead, soles of the feet, palms of the hands, scalp and solar plexus region	In the first article, pressure was applied manually with the fingertips, with each point receiving 2 min of pressure. In the second article, patients received pressure on the 5 points for 5 to 15 min for 2 consecutive days	Patients undergoing cardiac surgery and admitted to the ICU	Improved sleep quality, and one article reported a decrease in anxiety levels
Acupressure with valerian oil—2 articles (3.84%)			
2.5% valerian oil	In one of the articles, patients received bilateral massage on acupuncture points (Wind Pool, behind the head, on the glabella point, on the forehead, on the Shenmen of the ear, on the spirit gate of the wrist and on the Spring Well on the soles of the feet bilaterally), with 2 drops of oil for 2 min, for three nights. In the other article, patients received acupressure at the following points: Shenmen; Neiguan and Younquan for 3 min at each acupoint, totaling 18 min	Patients in coronary care units and general intensive care units	Significant improvement in sleep quality
Auriculopressure – 1 article (1.92%)			
Vaccaria seeds and adhesive for fixing	Disinfection of the five auricular points: Shenmen; sympathy; occiput; heart and anterior lobe, followed by fixation of the seeds with adhesive. The points were stimulated four times a day for 1 min with firm pressure, daily, 6 days a week, for 2 weeks	Adult patients undergoing cardiac surgery	Improvement in sleep quality. No difference in anxiety
Therapeutic touch – 1 article (1.92%)			
Performed in a calm environment, observing the patient's responses. Individual and standardised application	The intervention was applied to the hands with gentle, rhythmic movements and without pressure. Each session lasted 15 min, three times a week, for four consecutive weeks	Patients in palliative care	Significant improvement in sleep quality
Back massage—3 articles (5.76%)			
Use of oil and hot basalt stones, on a bed with privacy and adequate lighting, and manual massage	Three sessions were performed, lasting 10 min each; the oil was applied using slow, rhythmic, standardised movements and the hot stones were placed on the same points stimulated during the oil massage, with the stones finally being placed on the sacral points: T3 and T6. In another article: back massage using a standardised technique (techniques: circular movements; kneading; sliding with the thumb; tapping and friction) at 9 PM for 10 min, for 3 consecutive nights	Elderly women; adult men recovering from orthopaedic surgery; and ICU	Significant improvement in sleep quality and reduction in anxiety levels
Progressive muscle relaxation (PMR)—2 articles (3.84%)			
In one article: a CD, a brochure and a form were given to patients, who were instructed to practice twice a day for a month and fill out the form after each practice session. In another article, only an explanatory brochure was used	In the first article, patients were taught PMR in three 30‐min sessions. They were instructed to concentrate on each muscle group for 5 to 10 s (contracting and relaxing). In the second article, patients were individually taught, with the help of a brochure, to perform the exercises before bedtime, for 30 min, once a week (instructed to lie on their backs in a chair, with their arms at their sides and slightly away from their legs. Participants then tensed specific muscle groups for a few seconds and then relaxed them)	Haemodialysis patients and elderly residents of a long‐term care facility	Improved sleep quality
Benson relaxation technique (BR) and progressive muscle relaxation (PMR)—1 article (1.92%)			
The training sessions were conducted with the aid of a standardised CD containing music and instructions, which was given to participants at the end of the session. Participants also received the same model MP3 player and special headphones	An in‐person, individual training session was held to teach BRT techniques (focusing on calm nasal breathing and mentally repeating a word or phrase, allowing relaxation to occur naturally) and PMR (sequential contraction and relaxation of muscle groups, combined with deep breathing, to promote bodily relaxation). The intervention lasted 30 to 45 min, and patients were instructed to perform the exercises twice a day (20 min each time) for 4 weeks after hospital discharge	Patients undergoing cardiac surgery	Improvement in overall sleep quality
Progressive muscle relaxation (PMR) combined with deep breathing—1 article (1.92%)			
Training and practice of Jacobson's progressive muscle relaxation technique: sequential contraction and relaxation of muscle groups; deep breathing with nasal inhalation and oral exhalation; performing the technique in a supine position, in an undisturbed environment	After initial instruction, patients in the intervention group performed daily PMR sessions lasting 20 to 30 min for three consecutive days, always under the supervision of the researcher	Patients in the burn unit	Significant reduction in anxiety levels and improved sleep quality
Environmental interventions ‐ 7 articles (13.46%)			
In three articles, eye masks and/or ear protectors were used during the night. In addition, one article based its intervention on Jean Watson's Theory of Human Care	Closing of room doors at 11 PM; reduction of lighting; reduction of telephone and alarm volume; reorganisation of exams; nursing care and activities before 10 PM/11 PM or after 5 AM/6:30 AM; reduction of staff voice tone during the night, temperature control and humidity control. In some articles, patients received interventions for three consecutive nights (10 PM to 6 AM); for only one night (9 PM to 6 AM); during the night throughout the postoperative period; and throughout the hospital stay	Cardiac patients; in general, cardiac or surgical ICUs; hospitalized for certain cardiovascular surgeries; surgical patients	There was an improvement in sleep quality in most studies. The study based on Jean Watson's theory identified a significant reduction in anxiety
Guided imagery—1 article (1.92%)			
An instructional guided imagery CD was used, whose content was deep breathing, muscle relaxation and imagining pleasant scenarios (nature; sounds and images)	Sessions were conducted using the CD in a quiet environment. Each patient received a phone call to remind them to listen to the CD that day. This intervention was applied six times a week for 25 min over four consecutive weeks	Adults on chronic haemodialysis	Significant reduction in state and trait anxiety levels, as well as improved sleep quality
Aromatherapy massage—2 articles (3.84%)			
One of the articles used lavender essential oil diluted in almond oil (three drops of lavender oil and 15 mL of almond oil) and the other used only lavender essential oil	The first article performed back massage (on the patient's unburned area, including back surface stroking, back deep stroking, effleurage, re‐effleurage, back deep stroking and back surface stroking, with each step lasting 5 min) with the selected oil mixture, following six standardized steps, in a controlled, quiet environment with a standardized temperature (27°C). The second article performed topical application of 3–5 mL of oil with gentle circular movements after skin hygiene on the patient's bed	Women in the burn ward and adults admitted to the surgical ICU	Improved sleep quality and anxiety relief
Massage and Reiki—1 article (1.92%)			
During the massage, the Anmá protocol was applied, followed by Reiki	Anmá Protocol: kneading and pressure on the back, neck, chest, lower back, buttocks, thighs and feet (20 min). Reiki Protocol: considered the laying on of hands for 2–5 min on each of the following positions: eyes, occipital region, laryngeal region and sternum or cardiac region (total duration of 10 min). The sessions took place twice a week, totaling 8 sessions in 1 month of treatment	Volunteers at the outpatient clinic of the Institute of Integrated and Oriental Therapy (ITIO)	Significant reduction in state anxiety
Baduanjin exercises—1 article (1.92%)			
A video tape demonstrating the Baduanjin exercise and an educational leaflet with pictures and instructions on how to perform an exercise routine were used	The Baduanjin exercise included three stages: [1.] warm‐up stage, the participants relieved their tension and warmed up their joints. [2] Baduanjin exercise (steps: 1. Support the heaven with both hands regulate the three visceral cavities housing the internal organs; 2. Drawing a bow to each side resembles shooting an eagle; 3. Holding up a single hand regulates the spleen and the stomach; 4. Practising looking backward relieving the exhaustion; 5. Shaking the head and wagging the tail to remove excess heat from the heart; 6. Touching the feet with both hands to rid the heart of its illness; 7. Clenching fists and glaring to increase physical strength; 8. Shaking the body to ward off all illness). [3] Cool‐down stage, the participant could relieve their lactic acid accumulation and regulate their breathing pattern after exercise	Taiwanese seniors in the community who do not exercise	Significant improvement in overall sleep quality
Laughter yoga therapy—1 article (1.92%)			
Seven sessions in small groups (each with six seniors) held twice a week for 4 weeks. The sessions lasted 30 to 45 min and were conducted in a comfortable environment with plenty of natural light	The following was carried out: a brief 5‐min theoretical explanation of the benefits of laughter yoga therapy. This was followed by a 10‐min warm‐up with breathing and stretching exercises accompanied by background music; 10 min of guided playful laughter. Finally, there was 10 min of laughter meditation and anchoring techniques, which included guided body relaxation and deep breathing exercises	Elderly people living in the community	Reduction in anxiety levels and increase in happiness (subjective well‐being)
Spiritual nursing care, based on Swanson's theory of care and O'Brien's dimensions of spiritual care—1 article (1.92%)			
The intervention was carried out individually, at the bedside, following structured steps of spiritual care	Preparation of the environment (privacy, ventilation, lighting); Therapeutic presence (being with the patient, active listening, therapeutic silence); Verbal and nonverbal communication; Meeting basic needs; Facilitating spiritual/religious practices when necessary; Family involvement; Words of encouragement and motivation	Adults diagnosed with non‐hemorrhagic stroke	Significant reduction in anxiety levels in stroke patients
Deep breathing exercises—1 article (1.92%)			
In‐person training + written material (brochure)	The exercises took place after in‐person training every 3 h, beginning after hemodynamic stability post‐surgery. Exercises: Deep inhalation through the nose; breath retention for 2 to 5 s; slow exhalation through the mouth; 10 breaths per session	Adults undergoing cardiac surgery	Reduction in anxiety levels and improvement in sleep quality
Group counselling based on the Solution Focused Approach—1 article (1.92%)			
The intervention was applied to groups of 5 to 8 participants over 8 weeks, in weekly face‐to‐face sessions lasting approximately 90 min, with a 10‐min break after the first 45 min	Group counselling based on a solution‐focused approach, consisting of eight structured sessions, including: group awareness building; identification of individual resources and strengths; goal setting; solution‐focused questioning techniques; weekly homework assignments; reinforcement of positive behaviours; final assessment of perceived changes	Adults with eating disorders	Significant reduction in trait anxiety levels
Nursing intervention based on motivational interviewing—1 article (1.92%)			
Intervention consisting of three 40‐min motivational interviews during the 6 weeks prior to the surgical procedure	Patients received an individual, informative nursing session on surgical preparation and the procedure, followed by a motivational interview (based mainly on the participants setting their own goals for gradually changing their lifestyles)	Patients scheduled for knee replacement surgery	Reduction in anxiety levels
Nursing intervention: Sleep Improvement (NIC 1850)—1 article (1.92%)			
The intervention was integrated into the nursing care plan throughout the hospitalisation and was applied continuously and systematically by nurses, in accordance with the activities outlined in NIC 1850	Activities: assessment of sleep–wake patterns; sleep monitoring and recording; guidance on the importance of sleep; adjustment of the environment (light, noise, temperature); encouragement of sleep routines; control of stimuli; guidance on foods, beverages and medications that interfere with sleep; non‐pharmacological sleep induction techniques (muscle relaxation, affectionate touch, positioning, massage); grouping of care to reduce awakenings and education of patients and family members on sleep promotion techniques	Adults admitted to a mental health inpatient unit, diagnosed with disturbed sleep pattern (NANDA)	Significant improvement in sleep
Structured meaningful follow‐up—1 article (1.92%)			
Continuous monitoring by a companion chosen by the patient and the nursing professional, offering emotional support through visual and tactile presence during the pre‐, intra‐ and post‐endoscopy phases	Significant monitoring was ensured by the nurse at three points in time: (1) pre‐procedure (immediate and intermediate), (2) during the endoscopic procedure (until sedation), (3) post‐procedure (anaesthetic recovery), with systematic monitoring of psychological, physiological and biochemical variables	Adult patients undergoing upper gastrointestinal endoscopy	Significant reduction in anxiety levels throughout all stages of the procedure
Comprehensive nursing intervention – 1 article (1.92%)			
PowerPoint presentation and educational videos, combined with direct guidance and supervision of exercises	The intervention included: health education (use of thyme honey, dental care); counselling; relaxation techniques; mouth opening exercises; active and passive range of motion exercises; stretching exercises; posture maintenance; chin tucks and shoulder blade squeezes. The nursing intervention lasted 10–15 min and the educational intervention lasted 30 min, performed 9 to 10 times a day for 5 consecutive days	Adult patients in the postoperative period of oral cancer	Significant reduction in anxiety levels
Nursing intervention based on smart medical care associated with targeted care—1 article (1.92%)			
Targeted nursing plan, combined with the use of smart monitoring technology and psychosocial care during hospitalisation	The following measures were implemented: psychological counselling; positive psychological encouragement; adjustment of the ward environment; regular nurse–patient communication; guidance on relaxation; rest and adherence to treatment; and use of an intelligent sleep monitoring system	Patients admitted to the cardiology unit	Improved sleep quality
WILD 5 Wellness Programme (multicomponent self‐care intervention)—1 article (1.92%)			
The programme was structured in a workbook; initial guidance by video and telephone; daily recording of practices and self‐management by the participant	The intervention consisted of implementing five main areas: daily physical exercise, mindfulness/meditation, sleep hygiene, social connection, healthy nutrition and daily positive psychology exercises (HERO)	Adults with psychiatric diagnoses: depression, anxiety disorders, bipolar disorder, attention deficit hyperactivity disorder	Significant reduction in anxiety; improvement in sleep quality and mental well‐being
SLEEP Bundle Protocol—1 article (1.92%)			
Evidence‐based continuous improvement project, with training for nursing staff, routine application of the SLEEP Bundle in the ICU, monitoring through audits, and continuous feedback to adjust practices	This intervention consisted of: Daily sleep assessment; organization of night care (11 PM–6 AM); noise and light reduction; use of eye masks and earplugs; aromatherapy and music according to patient preference; staff education and training	Adults admitted to the surgical ICU	Significant improvement in sleep quality

*Source:* S1. Database.

Regarding the nursing process, only two (3.12%) articles addressed interventions related to a specific nursing diagnosis. The study by Kisvetrová et al. (2013) investigated the ‘Spiritual Support’ intervention from the Nursing Interventions Classification (NIC) for the NANDA‐I diagnosis ‘Death Anxiety’ in palliative care patients. The authors identified 29 activities performed by nurses, such as treating the individual with dignity, listening to feelings, being available for support, offering music or spiritual materials and praying with the patient.

These interventions were described as facilitators of patient dignity, active listening and the nurse's presence in care. Conversely, the main barriers identified were lack of time, organisational limitations, lack of privacy, the religious beliefs of the professionals and insufficient training in addressing spirituality (Kisvetrová et al. 2013).

Regarding spirituality, Aslan and Çetinkaya (2022) conducted a clinical trial with palliative care patients where the intervention consisted of therapeutic hand touch with a spiritual approach. Significant improvements were observed in both spirituality perception and sleep quality after 4 weeks.

Additionally, Laguna‐Parras et al. (2013) focused on the nursing intervention ‘Sleep Enhancement’ for the diagnosis ‘Disturbed Sleep Pattern’, developing activities such as guidance on the importance of sleep, environmental adjustment, use of non‐pharmacological techniques, care to reduce nocturnal awakenings and educational strategies for sleep promotion. The study demonstrated improvement in sleep quality and scores in the Nursing Outcomes Classification (NOC). The authors highlighted that these interventions are easily implemented in routine care, independent of medication use.

Regarding the seven selected review articles, four systematic reviews investigated interventions for sleep promotion, specifically barrier interventions (earplugs and eye masks) and environmental interventions (minimising nocturnal patient handling and silencing monitor alarms), alongside non‐pharmacological interventions like aromatherapy, relaxation, massage, acupuncture, music therapy and guided imagery (Bellon et al. 2021; Hweidi et al. 2024; Hu et al. 2015; Lee et al. 2025). These reviews showed outcome improvement but highlighted the heterogeneity of primary studies and recommended larger clinical trials with standardised interventions.

The systematic review by Chandrababu et al. (2019) showed anxiety reduction through foot and hand reflexology, while the two umbrella reviews analysed environmental interventions for ICU sleep (Bellon et al. 2022) and non‐pharmacological/environmental interventions for pre‐operative anxiety (Agüero‐Millan et al. 2023). Both acknowledged therapeutic potential but called for better standardisation and theoretical grounding.

Among other study designs, a methodological study in Canada developed individualised education strategies based on Intervention Mapping, implemented by nurses for elderly patients and carers. The research used the teach‐back technique to ensure understanding and was adapted to individual contexts. Interventions focused on sleep promotion during and after hospitalisation, including natural light exposure, daytime physical activity, sleep hygiene and stimulus control therapy (Sidani et al. 2022).

Couto's dissertation (2022) addressed sleep promotion in the elderly by developing an educational booklet on relaxing massage, grounded in Jean Watson's Theory of Human Caring. Although not clinically applied, the author highlighted its relevance for improving quality of life and reducing stress.

In the elderly context, Yuan and Yuan's (2021) cohort study showed that nurses implementing specific psychological care, including support and clarification based on emotional state, significantly reduced anxiety and depression symptoms in post‐percutaneous coronary intervention patients compared to routine care.

Among experimental studies that did not show significance in all outcomes, Akkaya et al. (2024) found that inhaled lavender aromatherapy improved pain and sleep in burn patients, but not anxiety. Similarly, Bang and Park (2020) observed sleep improvement with auriculopressure in cardiac surgery patients, but no significant effect on anxiety. Both studies recommended larger sample sizes.

In line with the objectives of this scoping review, although some studies did not use specific tools to assess the outcomes analysed, such as those by Sidani et al. (2022) and Kisvetrová et al. (2013), their inclusion was relevant for mapping nursing interventions related to sleep and spiritual support, respectively, broadening the understanding of care strategies potentially targeted at these outcomes.

Considering the diversity of interventions mapped in this scoping review, several mechanisms of action were identified under the parameters evaluated. However, relaxation was the mechanism most frequently mentioned as a possible explanation for the observed effects (see Data S1: Database). In addition, some studies reported changes in biomarkers, such as cortisol and melatonin, associated with stress response and sleep regulation (Bellon et al. 2021; Hweidi et al. 2024). These findings suggest that relaxation is the most recurrent cross‐cutting element among the interventions analysed and may be related to the improvements observed in anxiety and sleep parameters (Orsal et al. 2014; El‐Sayed et al. 2024).

To enhance rigour, Table 4 presents the PAGER model (Bradbury‐Jones et al. 2022) based on the critical analysis of the selected research, emphasising research patterns, gaps and recommendations for future studies on nursing care in anxiety, sleep and well‐being.

**TABLE 4:** Pager framework obtained from the analysis of selected articles.

Standards	Advances	Gaps	Evidence for practice	Research recommendations
Anxiety	Non‐pharmacological strategies have proven effective in pre‐ and post‐operative anxiety, palliative care, burn patients, ICUs, wards and at home	There is a lack of studies focussing on interventions for anxiety disorders, with research predominantly focussing on situational anxiety	Environmental and non‐pharmacological interventions have shown positive effects on anxiety	Conducting studies that address different types of anxiety, beyond situational anxiety, as well as research that develops and validates intervention protocols
Sleep	Several studies have addressed environmental control and the use of earplugs and eye masks in Intensive Care Unit (ICU). Non‐pharmacological therapies have also shown positive results for sleep	Absence of objective sleep measures (such as actigraphy and polysomnography) in most studies	Environmental and non‐pharmacological interventions have shown a positive effect on sleep, especially in the ICU	Need for studies in other clinical settings, with larger samples, as well as standardisation of interventions and inclusion of objective measures of sleep assessment
Well‐being	Self‐care interventions, the use of care technologies and Laughter Yoga have demonstrated a positive impact on well‐being	Only four studies addressed wellbeing, generally as a secondary outcome, with limited depth	To be determined by future research	Expansion of studies that assess general and subjective well‐being, preferably associated with anxiety
Nursing process	Interventions related to spirituality and sleep promotion were identified	Few nursing diagnoses, interventions and outcomes were identified for anxiety, sleep and well‐being. No care plans were found. Spirituality was addressed in only two studies and was restricted to the context of palliative care	To be determined by future research	Further studies are needed that integrate the nursing process into the standardisation of care for patients with anxiety, as well as research on spirituality in different clinical nursing contexts

## 5 Discussion

Most of the studies included in this review evaluated nursing interventions aimed at situational anxiety rather than specific psychiatric contexts or anxiety disorders. Examples include studies on post‐operative anxiety (Toprak et al. 2024), patients with burns (Akkaya et al. 2024) and individuals undergoing haemodialysis (Afshar et al. 2018). Consistent with previous evidence, addressing anxiety and sleep represents an essential dimension for promoting patient mental health across different clinical contexts (Scott et al. 2021).

The only identified study that dealt directly with anxiety disorders and psychiatric patients was by Rolin et al. (2019), in which nursing developed interventions such as daily physical exercise, mindfulness/meditation, sleep hygiene, strengthening social bonds, healthy eating and positive psychology exercises.

The results of this review indicate that sleep has been an outcome widely investigated by nursing, corroborating Silva, Souza, et al. (2024); Silva, Gilbertoni, et al. (2024), who highlight sleep and mental health as recurring themes in research, primarily due to the direct relationship between sleep disturbances and psychological health. Evidence suggests that insomnia, poor sleep quality and excessive daytime sleepiness are associated with various psychiatric disorders, such as depression, anxiety and bipolar disorders, as well as negative repercussions on general well‐being (Cox and Olatunji 2020; Degasperi et al. 2024). In this regard, the meta‐analysis by Scott et al. (2021) demonstrated that interventions effective in promoting sleep quality also have statistically significant effects on anxiety, depression and stress.

Regarding environmental interventions, these aim to modify the hospital environment to reduce or eliminate external stimuli such as noise, excessive light and interruptions related to nursing activities (Bellon et al. 2022). According to the studies included in this research, such interventions were applied predominantly in ICUs (Dave et al. 2013; Li et al. 2010) and in patients undergoing cardiac surgery (Babaii et al. 2015), with the purpose of improving sleep quantity and quality.

Concerning the anxiety outcome, only two studies evaluated the impact of environmental interventions on anxious symptoms (Azizoğlu et al. 2025; Demiray and Khorshid 2018), the latter of which did not identify a significant effect of the intervention in reducing anxiety levels. As for well‐being, no studies were found that measured the positive or negative impact of this set of interventions on this outcome.

In addition to environmental modifications, which require care organisation and effective communication with the patient, the nursing team, as protagonists in implementing care aimed at promoting sleep and well‐being and reducing anxiety, also assumes a central role in patient health education. This is evidenced by Silva, Souza, et al. (2024); Silva, Gilbertoni, et al. (2024), who state that nurses, due to their proximity to patients, are in a strategic position to educate, guide and empower people to adopt healthy behaviours. This educational role of nursing was demonstrated in the study by Debnath et al. (2024), which describes the development of an educational support intervention focusing on post‐operative guidance.

A significant amount of research using integrative and complementary therapies for the analysed outcomes was also observed. These practices value autonomy, person‐centred care and the uniqueness of the individual, recognising their capacity to promote their own health. Furthermore, these practices are also aligned with evidence‐based practice, prioritising safe and effective interventions (World Health Organization [WHO] 2025b). In this scenario, nursing integrates conventional approaches and complementary therapies in a contextualised manner, with responsibility and sustainability (Miranda and Vieira 2021; Santos et al. 2022).

For the promotion of mental health, it is fundamental that the nurse and patient maintain a relationship of trust, with continuous dialogue and clear information (Sousa et al. 2025). To this end, the use of integrative and complementary therapies strengthens this therapeutic bond, reducing the centrality of technical care and favouring a person‐centred approach that considers not only clinical symptoms but also the psychosocial and spiritual factors involved (Wickert et al. 2023; Leal et al. 2025).

As observed in this review, the theme of spirituality in nursing care is still under‐explored but has potential beyond the context of anxiety in palliative care. Spiritual care can be understood as intuitive and interpersonal attention, guided by the nurse's sensitivity to the transcendent dimension of life and the real needs of the patient, expressing their way of being and caring (Sawatzky and Pesut 2025).

However, the review by Cunha et al. (2022) highlights that the main gap in nursing practice in this area lies in training still centred on technical competencies, with little emphasis on humanistic aspects. This results in professional insecurity and a lack of preparation to address spiritual needs. Thus, the authors argue for the need for curricular restructuring, continuous training and an expansion of the debate on spirituality and religiosity.

Regarding the well‐being outcome, it is defined as the active pursuit of activities, choices and lifestyles that lead to a state of holistic health (Global Wellness Institute 2020). In this context, the relevance of nursing in promoting and preserving patient well‐being is evident, as these professionals occupy a strategic position in providing holistic care capable of addressing the physical, emotional, social and spiritual needs of individuals (Sousa 2024).

Despite being an outcome of high relevance for an individual's health in a hospital context and constituting an important field of action for the nursing team, well‐being was rarely addressed, measured or investigated in the selected articles. Few interventions in the studies included in this review were identified as being directed exclusively at its promotion. Among the selected articles, the study by El‐Sayed et al. (2024) stands out, which applied Laughter Yoga Therapy in older people, evidencing an improvement in the participants' subjective well‐being.

It was also observed that other interventions analysed across the studies had repercussions on well‐being by acting on related outcomes, such as reduced anxiety and improved sleep quality. For example, the study by Rolin et al. (2019), conducted among patients with psychiatric diagnoses, implemented a multimodal intervention including daily physical exercise, mindfulness/meditation, sleep hygiene, social connection, healthy nutrition and daily positive psychology exercises (HERO).

Furthermore, the incipience of research using the nursing process for these outcomes was evident in the studies included in this review, with few studies addressing nursing diagnoses, interventions and results (Laguna‐Parras et al. 2013; Kisvetrová et al. 2013). The nursing process is fundamental for professional autonomy and identity, as it systematises professional care, favours individualised and safe plans and promotes care centred on patient integrity (Gadelha et al. 2024). Thus, it is recommended to expand research in this area, especially regarding the standardisation of interventions, the use of nursing theories and the development of plans aimed at anxiety, sleep promotion and well‐being.

Based on the results of this review and the discussions presented, relevant recommendations for nursing research are highlighted, such as deepening themes that are still under‐explored, for example, spirituality and the nursing process in anxiety, as well as conducting multicentre studies that investigate the different identified interventions with more rigorous methodologies. This would reduce the heterogeneity between studies, as highlighted in some of the systematic reviews included in the present review. Furthermore, the need to expand investigations into anxiety disorders and the promotion of psychological well‐being is reinforced to strengthen nursing practice in mental health.

The limitations of this research include the absence of a methodological quality assessment of the studies and the heterogeneity of the analysed contexts, although all addressed anxiety, sleep or well‐being. Additionally, the unavailability of some full texts may have led to the loss of relevant evidence. As this is a scoping review, it is not possible to establish the causality or effectiveness of the interventions, although the presence of a significant number of systematic reviews and clinical trials among the included studies is noteworthy.

## 6 Conclusion

The results of this scoping review demonstrate that nursing care directed at anxiety, sleep quality and well‐being outcomes has been predominantly developed in the literature through complementary and integrative therapies. These include aromatherapy, music therapy, reflexology, acupressure, therapeutic touch, massage, relaxation techniques, Reiki and yoga.

Beyond these practices, other relevant care strategies were identified, including spiritual support and environmental interventions, such as the control of lighting, temperature and noise reduction. Furthermore, the use of guided imagery, group counselling and self‐care programmes highlights the potential of nursing in providing holistic and person‐centred care.

## 7 Relevance to Clinical Practice

This review presents, in a systematised manner, various types of interventions potentially applicable to the care of individuals with anxiety, as well as strategies aimed at promoting sleep quality and, to a lesser extent, well‐being. The availability of these interventions expands the nurse's clinical repertoire, encouraging the autonomous and evidence‐based use of care practices. Notably, most of the strategies identified can be incorporated into daily care without requiring high costs or major structural changes. Furthermore, they represent significant opportunities for the development of new research across diverse nursing practice contexts.

## Author Contributions

Louise Constancia de Melo Alves Silva, Samia Valeria Ozorio Dutra, Barbara Fernandes Ferreira, Rodrigo Assis Neves Dantas participated in the conceptualization of the study. Louise Constancia de Melo Alves Silva, Samia Valeria Ozorio Dutra, Barbara Fernandes Ferreira participated in the methodology, review and editing of the study. Yenifer Lizeth Gañan Rojas, Kauanny Vitoria Gurgel dos Santos, Carlos Jordão de Assis Silva, Hylarina Maria Montenegro Diniz Silva, Kátia Regina Barros Ribeiro, Daniele Vieira Dantas participated in the visualization of the study. Louise Constancia de Melo Alves Silva, Samia Valeria Ozorio Dutra, Barbara Fernandes Ferreira participated in the investigation of the manuscript. Samia Valeria Ozorio Dutra, Yenifer Lizeth Gañan Rojas, Kauanny Vitoria Gurgel dos Santos, Carlos Jordão de Assis Silva, Hylarina Maria Montenegro Diniz Silva, Kátia Regina Barros Ribeiro, Daniele Vieira Dantas, Rodrigo Assis Neves Dantas participated in supervision of the manuscript. All authors have read and approved the final version of the manuscript.

## Funding

This work was funded by the Coordination for the Improvement of Higher Education Personnel (CAPES), grant number: 88887.967810/2024‐00, linked to the Postgraduate Programme in Nursing at the Federal University of Rio Grande do Norte (UFRN).

## Conflicts of Interest

The authors declare no conflicts of interest.

## Supporting information


**Data S1:** Database.

**Data S2:** Preferred Reporting Items for Systematic reviews and Meta‐Analyses extension for Scoping Reviews (PRISMA‐ScR) checklist.

## Data Availability

The data that supports the findings of this study are available in the Supporting Information of this article.
